# Characterization of Bacterial Community Dynamics of the Human Mouth Throughout Decomposition *via* Metagenomic, Metatranscriptomic, and Culturing Techniques

**DOI:** 10.3389/fmicb.2021.689493

**Published:** 2021-06-07

**Authors:** Emily C. Ashe, André M. Comeau, Katie Zejdlik, Seán P. O’Connell

**Affiliations:** ^1^Department of Biology, Western Carolina University, Cullowhee, NC, United States; ^2^Integrated Microbiome Resource, Dalhousie University, Halifax, NS, Canada; ^3^Department of Anthropology and Sociology, Forensic Osteology Research Station, Western Carolina University, Cullowhee, NC, United States

**Keywords:** postmortem interval, human decomposition, 16S rRNA community profile, metagenomics, metatranscriptomics, accumulated degree days, bacterial cultures, oral thanatomicrobiome

## Abstract

The postmortem microbiome has recently moved to the forefront of forensic research, and many studies have focused on the idea that predictable fluctuations in decomposer communities could be used as a “microbial clock” to determine time of death. Commonly, the oral microbiome has been evaluated using 16S rRNA gene sequencing to assess the changes in community composition throughout decomposition. We sampled the hard palates of three human donors over time to identify the prominent members of the microbiome. This study combined 16S rRNA sequencing with whole metagenomic (MetaG) and metatranscriptomic (MetaT) sequencing and culturing methodologies in an attempt to broaden current knowledge about how these postmortem microbiota change and might function throughout decomposition. In all four methods, Proteobacteria, Firmicutes, Actinobacteria, and Bacteroidetes were the dominant phyla, but their distributions were insufficient in separating samples based on decomposition stage or time or by donor. Better resolution was observed at the level of genus, with fresher samples from decomposition clustering away from others *via* principal components analysis (PCA) of the sequencing data. Key genera in driving these trends included *Rothia*; *Lysinibacillus*, *Lactobacillus*, *Staphylococcus*, and other Firmicutes; and yeasts including *Candida* and *Yarrowia*. The majority of cultures (89%) matched to sequences obtained from at least one of the sequencing methods, while 11 cultures were found in the same samples using all three methods. These included *Acinetobacter gerneri*, *Comamonas terrigena*, *Morganella morganii*, *Proteus vulgaris*, *Pseudomonas koreensis*, *Pseudomonas moraviensis*, *Raoutella terrigena*, *Stenotrophomonas maltophilia*, *Bacillus cereus*, *Kurthia zopfii*, and *Lactobacillus paracasei*. MetaG and MetaT data also revealed many novel insects as likely visitors to the donors in this study, opening the door to investigating them as potential vectors of microorganisms during decomposition. The presence of cultures at specific time points in decomposition, including samples for which we have MetaT data, will yield future studies tying specific taxa to metabolic pathways involved in decomposition. Overall, we have shown that our 16S rRNA sequencing results from the human hard palate are consistent with other studies and have expanded on the range of taxa shown to be associated with human decomposition, including eukaryotes, based on additional sequencing technologies.

## Introduction

The period of time between death and when a body is discovered, also called the postmortem interval (PMI), can provide crucial information when conducting a medicolegal death investigation. There are many methods investigators have used to determine a PMI, including body temperature (algor mortis), rigor mortis, livor mortis, and stage of soft tissue decomposition as well as using more circumstantial evidence – such as when a person was last seen or the last time they checked their mail (Megyesi et al., 2005; Goff, 2010). Forensic entomology, a field of study which investigates the role that various insect species and other arthropods play in decomposition, has also been used in determining PMI (Goff, 2010). These methods can provide PMI estimates that range from hours to days, weeks, months, and even years, often depending on what state of decomposition the body was found (Megyesi et al., 2005; Swift, 2006; Goff, 2010). Due to how the body decomposes, more rapid changes (e.g., algor mortis, rigor mortis, and livor mortis) occur near the beginning of decomposition and can thus provide more accurate PMI ranges (Goff, 2010). However, the interpretation of these findings is often subjective (Madea and Henssge, 2015). As these intrinsic processes subside and other information must be used, the accuracy with which PMI can be estimated begins to decrease, resulting in much wider windows for the time of death (Megyesi et al., 2005; Swift, 2006; Goff, 2010; Madea and Henssge, 2015). Often this results in skeletonized remains having PMIs that range from a day to months to sometimes years (Megyesi et al., 2005).

Recently, numerous studies have centered on analyzing the utility of the human microbiome in more accurately determining the PMI. The intrinsic nature of the human microbiome and the large role the microbial community plays in the progression of decomposition makes microbial-based PMI estimation an appealing forensic technique because it would reduce the reliance on postmortem processes that are heavily influenced by external conditions, such as forensic entomology which can often yield broad PMI estimations especially in cases with extensive decomposition or cases where physical barriers delay insect activity (Goff, 2010; Johnson et al., 2013; Javan et al., 2016). The postmortem microbiome has even shown promise in providing more accurate PMI estimates for skeletonized remains through analysis of communities present in fresh and weathered bone (Damann et al., 2015). In addition to skeletal remains, other studies have focused on the postmortem microbial communities associated with a variety of soft tissue (Javan et al., 2016). Due to its ease of access, the bacterial community of the oral cavity has been a common target for many of these studies (Hyde et al., 2013, 2015; Javan et al., 2016; Adserias-Garriga et al., 2017). The postmortem oral microbial community has been shown to exhibit distinct differences between early and late stage decomposition and to exhibit a community that is distinct from other locations in the body but not significantly different between sexes (Hyde et al., 2013; Javan et al., 2016; Adserias-Garriga et al., 2017). The microbial community of the oral cavity has also been shown to include more environment-associated taxa and become less host-associated as decomposition progresses (Adserias-Garriga et al., 2017). Distinctions between early and late stage communities appear to be driven by the bloat stage and the host-associated Actinobacteria found in the oral cavity throughout early decomposition (Hyde et al., 2013; Adserias-Garriga et al., 2017).

Carter et al. (2015) investigated how different seasons (i.e., summer vs. winter) can influence the gravesoil microbial community composition. They demonstrated that even in soils not associated with a carcass, bacterial communities were markedly different between seasons (Carter et al., 2015). Specifically, they showed how gravesoil microbial communities were more stable in winter compared to communities observed in the summer. Bashan et al. (2016) demonstrated that in healthy living individuals, the gut and oral microbiomes share universal similarities between individuals while the skin is more likely to be influenced by the environment of the host. In death, this may not hold true for the oral cavity due to its increased exposure to the environment and the lack of the immune system of the host to mitigate community changes. Compared to internal organs and bones, the oral cavity is more exposed to the external environment, making it more susceptible to environmental influences such as weather and scavenging. This likely makes the bacterial community living within the oral cavity more susceptible to seasonal variation.

This study focused on examining the postmortem succession of the bacterial communities found in the oral cavities of three human donors. The communities were assessed using 16S rRNA gene sequencing (hereafter, abbreviated 16S), total metagenomic (MetaG), and metatranscriptomic (MetaT) analyses, and culturing of bacteria on solid media. Our overarching goals were to connect patterns of microbial diversity to PMI during human decomposition and to link cultures of bacteria to distinct stages and processes throughout decomposition. While none of these methods alone provides a complete picture of the bacterial assemblages, making connections between these approaches could elucidate dynamic interactions between populations and be used to estimate important benchmarks of the PMI.

## Materials and Methods

### Donors and Sample Collection

Three donors, one male and two females, were placed in the open-air Forensic Osteology Research Station (FOREST) at Western Carolina University, located in Cullowhee, NC (coordinates 35°18'34”N 83°11'52”W). The donors were all elderly and died of natural causes (Table 1) and all were unembalmed. Donor 1, a male, had dentures in place when they arrived while Donor 2, a female, had a full set of teeth with some metal components and Donor 3, a female, was edentulate. Upon their arrival at the facility, each donor was immediately unclothed and placed on the ground in a supine position with no barriers to wildlife or the elements employed. Donor 2 was received in an early stage of decomposition because she had been deceased in her home for 2 days prior to discovery. All donors had been stored in the cold after death for at least 6 days before being transported to FOREST. This storage was related to legal requirements of a certified death certificate to accept donated bodies and the scheduling of transportation of bodies from across the state of North Carolina. Multiple oral swabs of the entire hard palate were taken during each sampling event; one swab was collected for DNA extraction, one for RNA extraction, and one for culturing techniques. Each donor was sampled 5–7 times throughout decomposition, including swabbed samples taken upon deposition at the facility (Table 1). The sampling times did not overlap between the donors, although each donor was placed in FOREST within 1–3 days after the end of the sampling period of the previous donor. Samples were collected using sterile Puritan® Hydraflock flocked swabs with a 30 mm break point and dry transport tube (Puritan Medical Products, Guilford, ME, United States). Generally, swabs were individually moistened in the field using separate aliquots of sterile molecular biology grade water prior to sample collection. The swabs intended for nucleic acid extraction were placed back in their tubes and immediately frozen on dry ice. The swabs intended for culturing were broken off on-site into a 2-ml microcentrifuge tube of sterile 15% glycerol/R2B medium (recipe is below in the Bacterial Isolates section) and placed on dry ice. The samples were immediately transported back to the lab where they were stored at −80°C.

**Table 1:** Information and sampling schedule for three human donors undergoing decomposition at the Forensic Osteology Research Station (FOREST) facility in Cullowhee, NC.

Donor information	Date of death/Cause of death	Sampling date	Sampling event	ADD	Decomposition state	Insect activity
ID: D1	4/03/2018	4/09/2018	1	0	Fresh	0
Sex, Age: M, 65	Gastric cancer	4/13/2018	2	49	Early	2
Height: 180 cm		4/16/2018	3^*^	89	Early	1
Weight: 54.4 kg (BMI = 16.7)		4/20/2018	4	138	Early	2
Ethnicity: White		4/23/2018	5^*^	168	Advanced	0
		4/27/2018	6^*^	222	Advanced	1
		4/30/2018	7	253	Advanced	0
ID: D2	4/23/2018	5/01/2018	1	41	Early	1
Sex, Age: F, 77	Heart disease	5/04/2018	2	106	Early	3
Height: 160 cm		5/07/2018	3	155	Early	2
Weight: 74.8 kg (BMI = 29.2)		5/11/2018	4^*^	223	Advanced	1
Ethnicity: White		5/14/2018	5	292	Advanced	1
ID: D3	5/11/2018	5/17/2018	1^*^	0	Fresh	0
Sex, Age: F, 84	Pneumonia	5/21/2018	2^*^	84	Early	3
Height: <140 cm		5/24/2018	3^*^	169	Advanced	2
Weight: <60 kg (BMI < 25)		5/28/2018	4^*^	291	Skeletonization	1
Ethnicity: White		6/01/2018	5^*^	392	Skeletonization	0

The first sampling event coincided with when the donors were received and placed at the facility. Samples consisted of oral swabs taken from the hard palate of each donor. ADD are reported for each sampling event as is a qualitative assessment of the decomposition state (based on Megyesi et al., 2005). Insect activity is reported as: 0 = no visible insect activity, 1 = only adult flies present, 2 = adult flies and small maggots present, and 3 = large quantities of both adult flies and maggots present. (Note: For Donor 3, height and weight were not reported and upper estimates are shown.).

* Samples were taken when it was raining (trace amounts of snow was present on 4/16/2018).

### Accumulated Degree Days

Located in the southern Appalachian Mountains, Cullowhee is a temperate climate (Cfa) that is classified as “fully humid with hot summers” (Peel et al., 2007). Because of the climate type and time of year, the donors were subjected to a wide range of environmental influences despite sampling spanning only 2 months. The facility experienced temperatures ranging from −1.7 to 32.2°C and received a total of 12.12 inches of precipitation during this time.^1^ Due to this variation, accumulated degree days (ADD) were used to standardize the decomposition process between donors (Table 1). ADD was calculated using the temperature measurements taken from iButton Thermochron® data loggers (Maxim Integrated, San Jose, CA, United States) that were placed in holders and staked next to the head of each donor. The iButtons were set to record the temperature every 30 min, resulting in 48 reads each day. This temperature data were then used to calculate a specific ADD for each sampling time using the following formula:

$$ADD_{t}=\overset{d_{x}-1}{\underset{d=0}{\sum }}\overset{-}{T}+\left(\frac{\sum_{s=0}^{t}\;T}{t}\right).\frac{t}{48}$$

Equation 1 Calculation of ADD. Variables are assigned as follows: *t* refers to the number of temperatures that were recorded prior to sampling on a given day (*d*), *d_x_* is the day on which the sampling occurred, *T* is the temperature in Celsius, and *s* refers to the number of iButton temperature records (48 total per day).

Since Donor 2 was received in an early state of decomposition, their starting decomposition measurement could not be zero and was instead estimated based on the National Institute of Health’s National Institute on Aging (NIA) recommendation for thermostat settings for the elderly during winter months (Calvin, 2014). According to the NIA, thermostats should be kept at an average of 69°F (20.5°C). Across 2 days, this resulted in a baseline of 41 ADD for Donor 2. Data collected from the field also included a qualitative estimate of the state of decomposition ranging from fresh, early, advanced, and skeletonization stages (Megyesi et al., 2005). Qualitative data were also recorded for insect activity for each of the donors throughout decomposition.

### Nucleic Acid Extraction and Purification

Samples were thawed at 4°C prior to nucleic acid extractions for 16S rRNA gene sequencing, whole genome shotgun MetaGs, and whole genome shotgun MetaTs. DNA and RNA were both extracted and purified using QIAGEN’s RNeasy® PowerMicrobiome® Kit (QIAGEN, Valencia, CA, United States) following the manufacturer’s protocol. The step involving phenol/chloroform/isoamyl alcohol was not utilized and dithiothreitol (DTT) was used in lieu of β-mercaptoethanol at a ratio of 20 μl of 2 M DTT per 1 ml of lysis buffer. During the third step of both DNA and RNA extractions, samples were placed in a BioSpec Mini-BeadBeater-1 (BioSpec, Bartlesville, OK, United States) at 2,500 rpm for 1 min. During RNA extraction, 70% ethanol was used in lieu of buffer PM4 during the addition of binding salts (buffer PM3) to prevent copurification of small RNAs. DNA extracts were stored at −20°C and RNA extracts at −80°C until sequencing or complementary DNA (cDNA) synthesis could be performed. cDNA was generated using Thermo Fisher Scientific’s SuperScript IV First-Strand Synthesis Kit (Thermo-Fisher Scientific, Waltham, MA, United States) following the manufacturer’s protocol before sequencing.

### Quantitation of Nucleic Acids

Nucleic acids were quantified using a 2100 Bioanalyzer (Agilent Technologies, Santa Clara, CA, United States). DNA was quantified using the Agilent DNA 12000 Kit and the DNA 12000 Series II Assay following the protocol detailed in the DNA 12000 Kit Quick Start Guide (Agilent Technologies) while RNA was quantified using the Agilent RNA 6000 Pico Kit and the Prokaryotic Total RNA Pico Series II Assay following the protocol detailed in the RNA 6000 Pico Kit Quick Start Guide (Agilent Technologies). One DNA sample (Donor 3, sample 3) was concentrated prior to sequencing by drying down the sample, then reconstituting it to half the original volume. cDNA was quantified using Invitrogen’s Qubit® 2.0 Fluorometer with the Qubit® dsDNA HS Assay Kit (Life Technologies, Carlsbad, CA, United States) following the protocol described in the Qubit® 2.0 Fluorometer User Manual.

### Library Preparation and Sequencing

Library preparation and sequencing were performed at the Integrated Microbiome Resource (IMR), located at Dalhousie University in Halifax, Nova Scotia. Libraries of the V6–V8 hypervariable region of the 16S rRNA gene were prepared using bacteria-specific primers (B969F = ACGCGHNRAACCTTACC; BA1406R = ACGGGCRGTGWGTRCAA) as outlined in Comeau et al. (2017) and sequenced on an Illumina MiSeq using 2 × 300 bp v3 chemistry. MetaG and MetaT libraries were prepared from extracted DNA or synthesized cDNA using the Illumina Nextera Flex kit (Illumina® Inc., San Diego, CA, United States), as per the manufacturer’s instructions, and sequenced on an Illumina NextSeq 550 using 2 × 150 bp HiOutput v2.5 chemistry.

### 16S rRNA Gene Sequence Data Analysis

Raw FASTQ files were demultiplexed on-instrument and then processed to create amplicon sequence variants (ASVs), as described in Comeau et al. (2017) and Nearing et al. (2018), using a custom SOP developed at the IMR as part of the continually evolving MicrobiomeHelper repository. The exact version of the analysis pipeline used is available at github.com/LangilleLab/microbiome_helper/wiki/Amplicon-SOP-v2-(qiime2-2019.7). In summary, custom scripts and the QIIME2 program (Bolyen et al., 2019) were used to perform read quality-control, denoising into ASVs using Deblur, and assignment of taxonomy against the SILVA reference taxonomy.

### Whole Metagenomic and Metatranscriptomic Data Analysis

Similar to above, raw FASTQ files were demultiplexed on-instrument and then processed using a pipeline under development at the IMR as part of the MicrobiomeHelper repository, the current version of which is available at github.com/LangilleLab/microbiome_helper/wiki. In summary, raw reads were quality-controlled using KneadData v0.7.2^2^, which employs Trimmomatic v0.36 (Bolger et al., 2014; options: SLIDINGWINDOW:4:20 MINLEN:50) and Bowtie2 v2.2.3 (Langmead and Salzberg, 2012; options: –very-sensitive –dovetail) to filter low-quality reads and screen out potential contaminant sequences against the human (GRCh38) and phiX174 genomes. “Raw” taxonomic composition was determined using Kraken2 v2.0.8 (Wood et al., 2019; option: –confidence 0.1), based upon a 150mer database built from the entire NCBI RefSeq Complete v93 database, and final taxonomic abundance profiles were generated using Bracken v2.0 (Lu et al., 2017; option: -t 10). Custom PERL and Python scripts (available above) were then used to do functional mapping of reads using MMseqs2 (Steinegger and Söding, 2017) against the entire UniRef90 database^3^, select the top hit for each read, map functions to Enzyme Commission (EC) numbers, and then generate either unstratified (function-only) or stratified (linking the above Kraken2 taxonomy) matrices. Two PICRUSt2 v2.2.0-b scripts (Douglas et al., 2020) were used to append functional descriptions (add_descriptions.py) to the above matrices and to generate KEGG pathway coverages (pathway_pipeline.py). Final functional coverages were normalized to reads-per-kilobase-million (RPKM). Processes were parallelized using GNU parallel (Tange, 2018).

### Bacterial Isolates

All cultures were grown and isolated in fall 2018 from frozen swab materials that were vortexed in sterile Reasoner’s 2A Agar (R2A formulation: 0.5 g yeast extract, 0.5 g proteose peptone, 0.5 g casamino acids, 0.5 g dextrose, 0.5 g soluble starch, 0.3 g sodium pyruvate, 0.3 g dipotassium phosphate, 0.05 g magnesium sulfate, and 15.0 g agar in 1 L distilled water) broth media without agar added (R2B), diluted, and spread plate onto either R2A plates (full strength or 1%) or 1% nutrient broth and 1% brain heart infusion agar media. These cultures were incubated at room temperature under a normal atmosphere. In total, 69 cultures were isolated, 46 of which were identified as distinct species. Fifty-three isolates were grown on R2A, while 16 isolates were cultured on the diluted media. The dilute media formulations were used in order to obtain slower growing bacteria that could have been active in decomposition or numerically abundant but difficult to detect otherwise. Once isolated, a variety of phenotypic tests were performed in order to characterize the species (Ashe, 2019).

Isolates were identified by sending cultures to GENEWIZ (GENEWIZ Inc., South Plainfield, NJ, United States) for DNA extraction and Sanger sequencing of the full 16S rRNA gene. Sequences were analyzed using BLAST in order to identify the closest sequence match for each culture (NCBI Resource Coordinators, 2016). Search parameters included blastn of 16S rRNA genes for sequences from type material and a cutoff value of 97% identity to designate an OTU. NCBI taxonomy designations were used to compare cultures to ASVs from the 16S rRNA gene sequencing and reads obtained in the MetaG and MetaT datasets.

The 16S rRNA sequences from the bacterial cultures were analyzed with the 328 ASVs (~400 bp in length) obtained from the 16S environmental sequencing. The ASVs and culture sequences were aligned using Clustal Omega followed by construction of a cladogram to group similar sequences (Madeira et al., 2019). Sequences that appeared to be closely related were compared pairwise by using BLAST and those that were ≥97% identical were considered to be the same OTU. Bracken was used to generate data to the species level for the MetaG and MetaT sequences. These data were then used to compare to OTUs from the cultured bacteria.

### Statistical Analyses

Daily precipitation and temperature data between donors were analyzed using one-way ANOVA and Bonferroni *post hoc* testing using Systat 10 for Windows (SPSS, Inc., Chicago, IL). Shannon diversity (H) and evenness (E_H_) of ASVs, MetaG, and MetaT reads at the species level were calculated using data that had been normalized to the sample with the lowest total number of sequences. These data were examined for statistical significance by grouping samples by donor, ADD, and decomposition stage (grouped by fresh + early vs. advanced + skeletonized) using ANOVA or *t* tests as above.

Principal components analysis was performed on data at the level of phylum and genus using BioVinci version 3.0.9 (BioVinci, Inc., San Diego, CA, United States) for each of the three sequencing methods. Proportional data were arranged by donor and sampling event for each donor across the taxonomic categories derived from the bioinformatics tool applied for each sequencing method. Vectors were also generated from the statistical output to indicate which taxa were most influential in separating the samples on the PCA plots.

## Results

### Climate and Other Factors Affecting Decomposition

**Figure 1:**
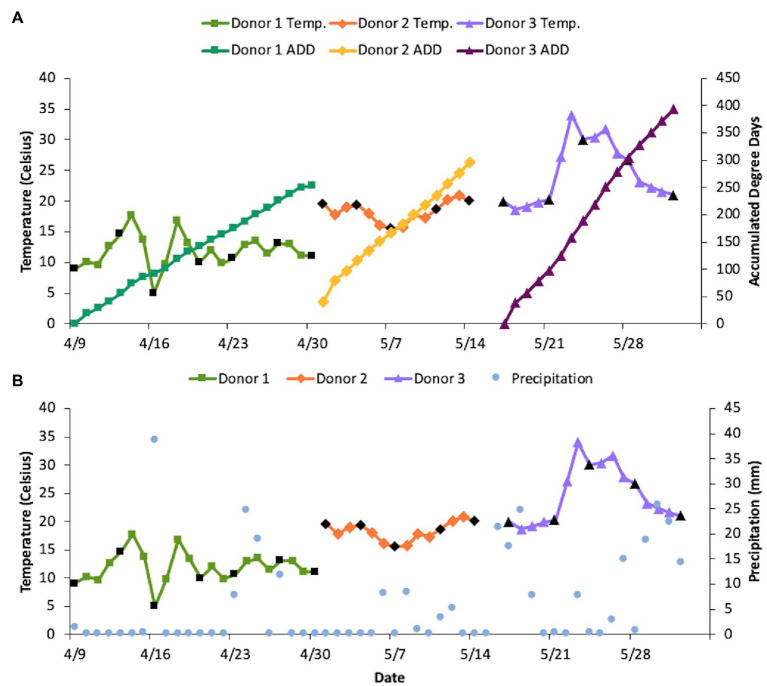
Differences in sampling period, temperature, precipitation (mm), and accumulated degree days (ADD) between donors. Black points denote days on which samples were collected. (Note: The last collection day for each donor was not able to be included because the temperature loggers were collected during sampling, therefore an average daily temperature could not be recorded for those days). **(A)** ADD compared to the average daily temperatures for each donor. Daily temperatures were significantly different between donors based on one-way ANOVA and Bonferroni *post hoc* test (*p* < 0.01 between all pairs of donors). **(B)** Comparison of net daily precipitation and average daily temperature for each donor throughout the sampling period. Precipitation was not significantly different between donors based on one-way ANOVA (*p* = 0.08).

The three donors in this study were elderly and died from different natural causes (Table 1). Estimated body mass indices (BMI) ranged from underweight for Donor 1 to almost obese for Donor 2, with Donor 3 estimated to be on the low end of the BMI scale. No official height or weight data were available for Donor 3. The donors were placed into the decomposition facility sequentially with none of their sampling periods overlapping one another, therefore exposing them to disparate temperature regimes (Figure 1). Snow was observed on 16 April, which also had the coldest recorded average daily temperature. Net daily precipitation was not significantly different between any of the donors (*p* = 0.08; one-way ANOVA), with Donor 1 seeing 0–38.6 mm precipitation each day, Donor 2 receiving 0–8.4 mm rain, and Donor 3 ranging from 0 to 25.7 mm of daily rain. The mean daily temperatures were significantly different for the three donors with temperatures increasing with the placement of each donor (*p* < 0.001 for one-way ANOVA and *p* < 0.01 for pairwise comparisons using the Bonferroni *post hoc* test; Figure 1A). The average daily temperatures ranged from 4.9–17.7°C (*x* = 11.8°C), 15.5–20.8°C (*x* = 18.3°C), and 18.6–34.0°C (*x* = 24.6°C), for Donors 1, 2, and 3, respectively, resulting in higher ADD values with each subsequent donor placed in the FOREST. The slopes for ADD over time were also found to be significant (*p* < 0.001). Donor 2 was placed in the facility at an estimated ADD of 41 while the others were included in the study at an ADD value of 0 after being stored cold after death for at least 6 days. The last sample for Donor 1 was taken at an ADD of 253, for Donor 2 at 292, and for Donor 3 at an ADD of 392. Donor 3 was the only of the three to reach the skeletonization stage of decomposition and, along with Donor 2, saw high amounts of insect activity throughout decomposition (Table 1). Additionally, camera trap footage and visible signs on the bodies revealed scavenging activity from vultures for all three donors.

### Diversity Measures From the Three Sequencing Approaches

Sequencing of the samples from the three donors yielded data for all 17 samples for the three sequencing methods used. The total number of 16S sequences ranged from 1,229 to 8,594 with an average of 4,822 (Supplementary Table 1). These yielded a total of 328 unique ASVs, with single samples ranging from 7 to 74 ASVs each and Donor 1 having significantly fewer ASVs on average than Donors 2 and 3 (one-way ANOVA *p* = 0.008; Bonferroni test *p* < 0.05). MetaG sequencing produced 1.3 × 10^5^–4.0 × 10^6^ total reads at the taxonomic level of species (*x* = 2.4 × 10^6^ reads) and 183–6,976 distinct species per sample from a total pool of 11,504 species. MetaT sequencing resulted in a range of 4.7 × 10^4^–1.8 × 10^6^ total reads at the level of species (*x* = 3.8×10^5^ reads), a total pool of 1,797 distinct species, and 46–746 species for each sample. There were no significant differences for Shannon diversity (H) or Shannon evenness (E_H_) for the 16S data based on neither ANOVA tests for data grouped by either donor or ADD range grouped into three classes (early, middle, and late) nor for *t* tests based on the fresh + early vs. the advanced + skeletonized stages of decomposition (Supplementary Table 1). The only significant differences seen were for MetaG data for E_H_ and MetaT data for H when comparing the three donors, with Donor 1 showing lower values than Donor 2, but neither showing differences from Donor 3 (one-way ANOVA *p* = 0.02; Bonferroni test *p* < 0.05, for both comparisons).

### 16S Taxonomic Distributions

**Figure 2:**
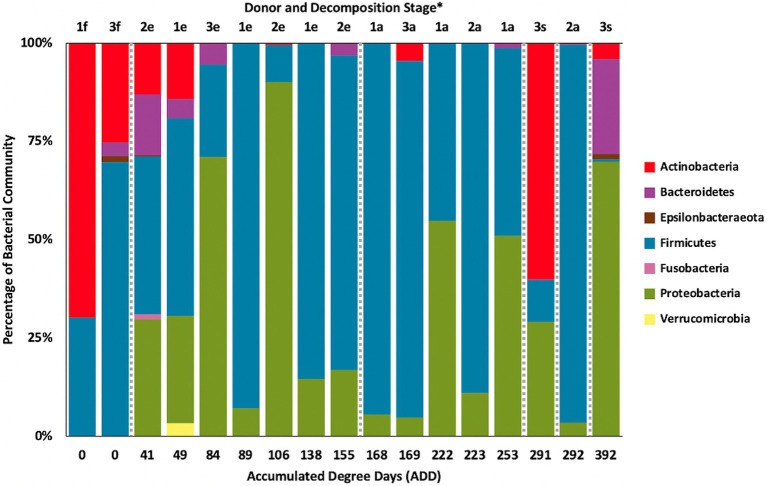
Proportion of bacterial phyla determined from 16S rRNA gene sequencing of the V6–V8 hypervariable region from oral samples of three human donors (Donors 1, 2, and 3) during decomposition. Decomposition was assessed as ADD on the *x*-axis, and samples were also classified to an overall decomposition stage for each donor at each sampling event (^*^f, fresh; *e*, early; *a*, advanced; and *s*, skeletonized).

**Figure 3:**
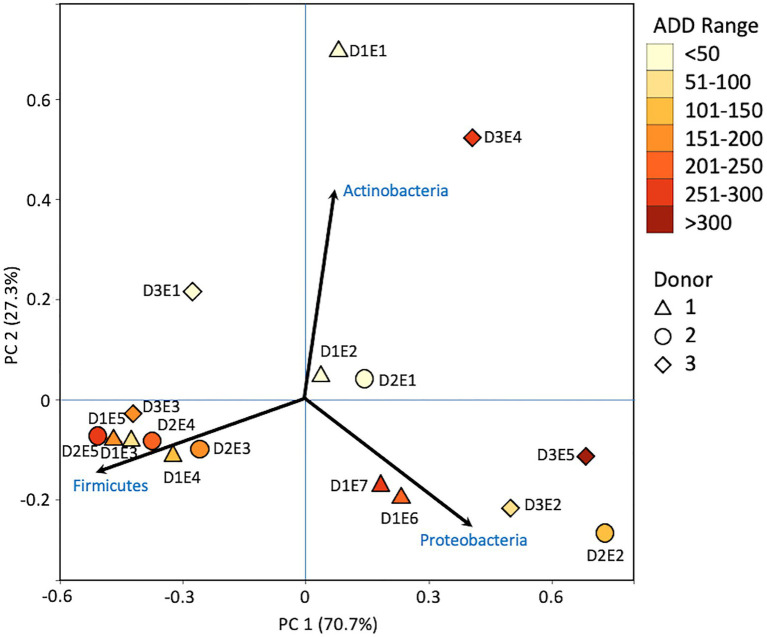
Principal components analysis (PCA) plot for the proportional data of bacterial phyla observed from 16S rRNA gene sequencing of the V6–V8 hypervariable region from oral samples of three human donors (Donors 1, 2, and 3) during decomposition. Shading is added to the symbols [triangles for Donor 1 (D1), circles for Donor 2 (D2), and diamonds for Donor 3 (D3)] to show earlier (lighter shading) to later (darker shading) ADD and also times sampled (from E1 to E7 for D1 and E1 to E5 for D2 and D3, indicating first sample to last sample taken).

**Figure 4:**
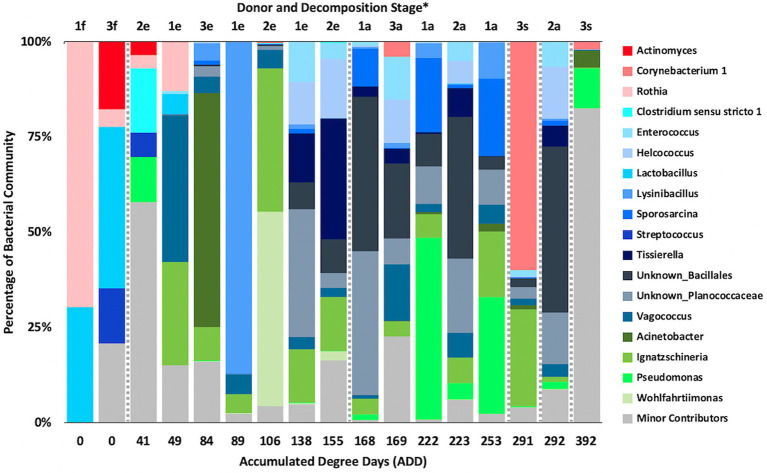
Proportions of bacterial genera determined from 16S rRNA gene sequencing of the V6–V8 hypervariable region from oral samples of three human donors (Donors 1, 2, and 3) during decomposition. Decomposition was assessed as ADD on the *x*-axis and samples were also classified to an overall decomposition stage for each donor at each sampling event (^*^f, fresh; *e*, early; *a*, advanced; and *s*, skeletonized). Minor contributors include data that accounted for <1% of the total diversity within a group.

**Figure 5:**
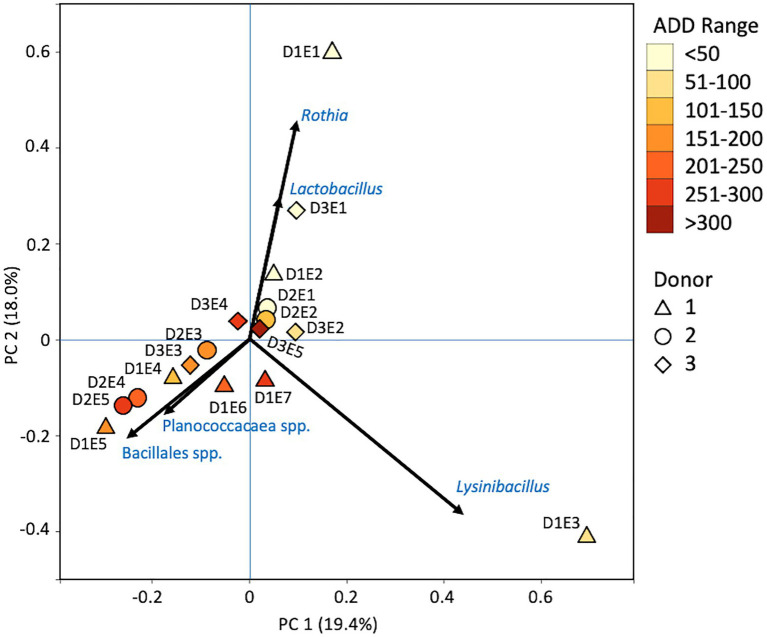
Principal components analysis plot for the proportional data of bacterial genera observed from 16S rRNA gene sequencing of the V6–V8 hypervariable region from oral samples of three human donors (Donors 1, 2, and 3) during decomposition. Shading is added to the symbols [triangles for Donor 1 (D1), circles for Donor 2 (D2), and diamonds for Donor 3 (D3)] to show earlier (lighter shading) to later (darker shading) ADD and also times sampled (from E1 to E7 for D1 and E1 to E5 for D2 and D3, indicating first sample to last sample taken).

Firmicutes dominated or co-dominated the samples collected from the donors based on the 16S sequencing, while the Proteobacteria were also found in sizable numbers in most samples and Actinobacteria were most numerous in two of the samples (Figure 2). PCA of these data revealed strong trends in separating samples based on Actinobacteria and Proteobacteria vs. Firmicutes with 70.7% of the variation in the dataset being explained by PC1 (Figure 3). Grouping of the samples was based on these phyla and no clear trends were seen based on donor or ADD, though the four earliest time points sampled were separated from almost all of the other samples based on PC2 which accounted for 27.3% of the variation. The diversity within each phylum was high for most samples and included many genera that each contributed to less than 1% of the total data (Figure 4). This latter case is evident for the first sample for Donor 2 and the final sample for Donor 3. *Rothia* spp. and *Lactobacillus* spp. were especially common during early decomposition and this can be seen in the PCA plot of the data in Figure 5, which separated early samples based on a combination of PC1 (19.4% of variance) and PC2 (18% of variance). *Lysinibacillus* spp. were only observed in large numbers (85% of the ASVs) in the third Donor 1 sample while *Streptococcus* spp., Bacillales spp., and Planococcaceae spp. were common among middle to late ADD samples. These latter two groups were influential in separating samples based on PC1 which also skewed the third sample for Donor 1, based on the high numbers of *Lysinibacillus* (Figure 5). *Pseudomonas* spp. was most common in the later samples.

### MetaG Taxonomic Distributions

**Figure 6:**
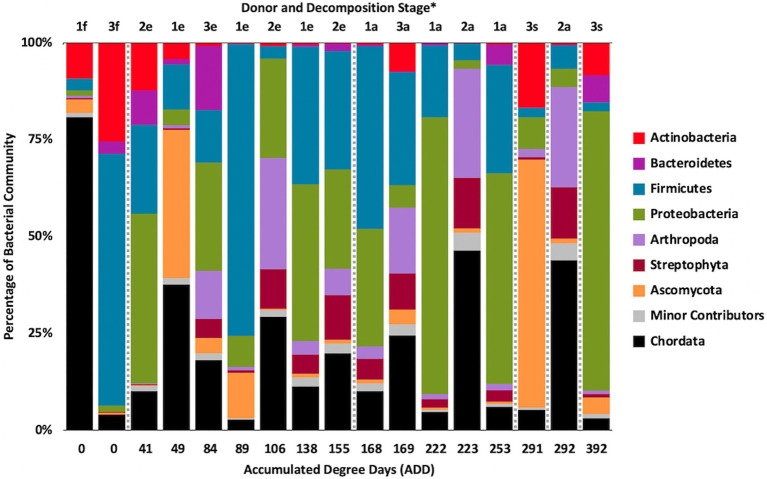
Proportions in percentages of bacterial and eukaryotic phyla determined from shotgun metagenomic (MetaG) sequencing from oral samples of three human donors (Donors 1, 2, and 3) during decomposition. Decomposition was assessed as ADD on the *x*-axis and samples were also classified to an overall decomposition stage for each donor at each sampling event (^*^f, fresh; *e*, early; *a*, advanced; and *s*, skeletonized). Minor contributors include data that accounted for <1% of the total diversity within a group.

**Figure 7:**
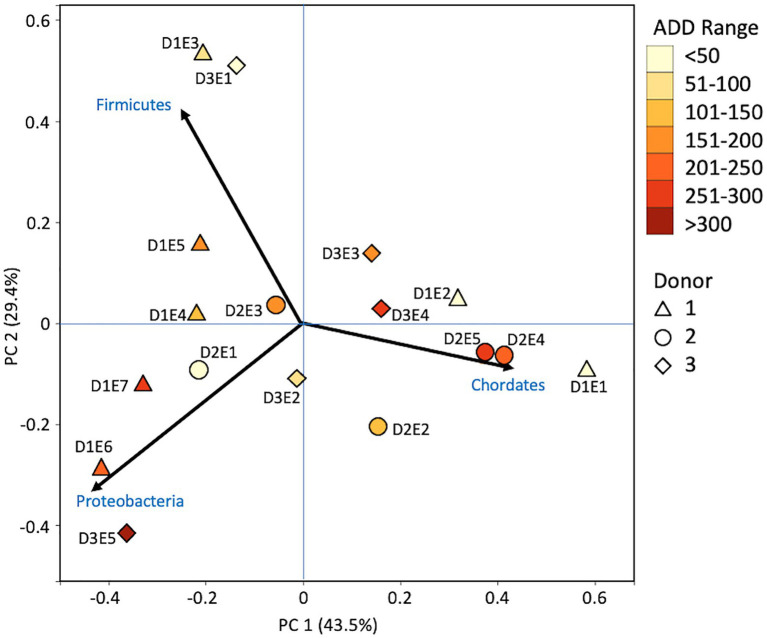
Principal components analysis plot for the proportional data of bacterial and eukaryotic phyla observed from shotgun MetaG sequencing from oral samples of three human donors (Donors 1, 2, and 3) during decomposition. Shading is added to the symbols [triangles for Donor 1 (D1), circles for Donor 2 (D2), and diamonds for Donor 3 (D3)] to show earlier (lighter shading) to later (darker shading) ADD and also times sampled (from E1 to E7 for D1 and E1 to E5 for D2 and D3, indicating first sample to last sample taken).

Metagenomic sequencing included reads for Chordata, Arthropoda, Ascomycota, and Streptophyta in addition to bacterial phyla (Figure 6). Three eukaryotic phyla were represented in most samples, with high numbers seen for Chordata for samples from Donors 1 and 2, for Arthropoda for Donor 2, and for Ascomycota in one sample each for Donor 1 and Donor 3. Streptophyta were common (>1% of the total samples) in 11 of the 17 samples. The Firmicutes and Proteobacteria were in the majority for bacteria in most samples, with higher numbers of Firmicutes being found generally in earlier samples and Proteobacteria being in higher amounts in later samples. Actinobacteria were primarily found in early and late samples, comprising up to ~25% of the community in some samples, but were generally less common. Chordates appeared to help drive the largest trend in the dataset, with Chordate-heavy samples separating in PCA from samples with high abundances of Firmicutes and Proteobacteria, based on PC1 which accounted for 43.5% of the total variation (Figure 7). Samples with high numbers of Firmicutes and Proteobacteria were separated based on PC2 (29.4% of total variation). Samples did not appear to cluster based on donor or ADD.

**Figure 8:**
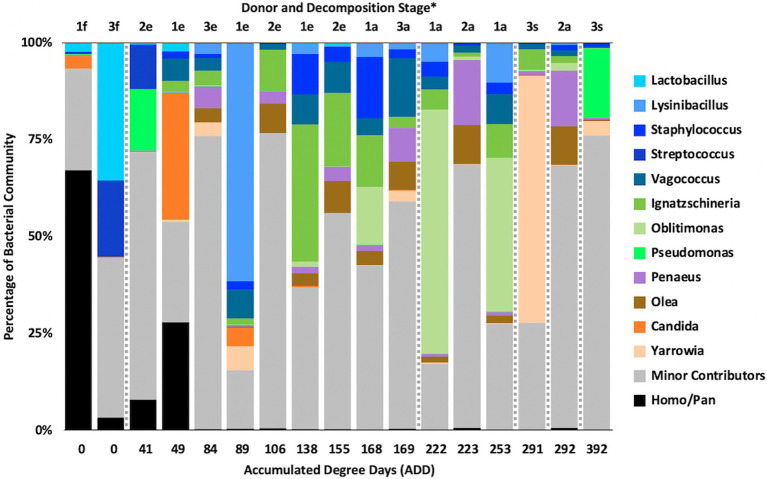
Proportions of bacterial and eukaryotic genera determined from shotgun MetaG sequencing from oral samples of three human donors (Donors 1, 2, and 3) during decomposition. Decomposition was assessed as ADD on the *x*-axis and samples were also classified to an overall decomposition stage for each donor at each sampling event (^*^f, fresh; *e*, early; *a*, advanced; and *s*, skeletonized). Minor contributors include data that accounted for <3% of the total diversity within a group.

**Figure 9:**
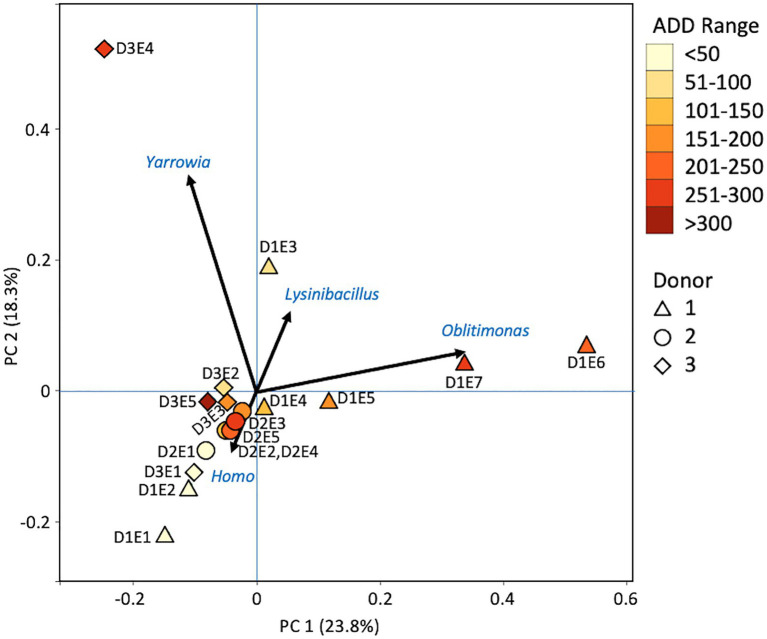
Principal components analysis plot for the proportional data of bacterial and eukaryotic genera observed from shotgun MetaG sequencing from oral samples of three human donors (Donors 1, 2, and 3) during decomposition. Shading is added to the symbols [triangles for Donor 1 (D1), circles for Donor 2 (D2), and diamonds for Donor 3 (D3)] to show earlier (lighter shading) to later (darker shading) ADD and also times sampled (from E1 to E7 for D1 and E1 to E5 for D2 and D3, indicating first sample to last sample taken).

At the level of genus, MetaG samples showed high diversity, with ~15–75% of the data for each sample attributed to minor contributors, which each individually accounted for <3% of the total reads (Figure 8). DNA from the human donors (*Homo* and the misidentified genus *Pan*) was common in the early samples as were reads from Firmicutes, including *Lactobacillus* spp., *Lysinibacillus* spp., and *Staphylococcus* spp., while some samples included *Candida* spp. Middle samples were richer in *Ignatzschineria* spp. and later samples contained more *Oblitimonas* spp. *Yarrowia* spp. dominated one of the last samples for Donor 3 and was common only in that donor. *Penaeus* spp. (an arthropod group), *Olea* spp. (plants), and *Vagococus* spp. were found throughout the samples in generally lower amounts. *Oblitimonas* spp. and *Lysinibacillus* spp. primarily separated the five middle to late Donor 1 samples from all others in PCA, based on PC1 (23.8% of the variation) while PC2 (18.3% of the variation) separated the fourth sample from Donor 3 based on *Yarrowia* spp. (Figure 9). The human reads were influential in separating the earliest samples from all others based on PC1 and PC2.

### MetaT Taxonomic Distributions

**Figure 10:**
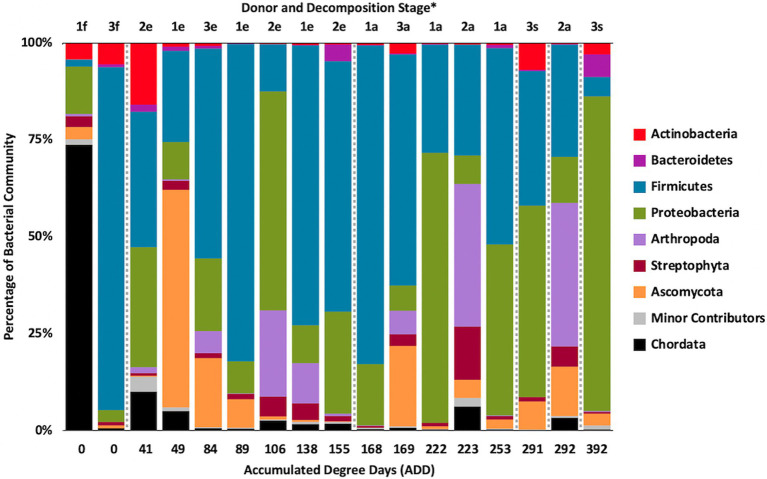
Proportions of bacterial and eukaryotic phyla determined from shotgun metatranscriptomic (MetaT) sequencing from oral samples of three human donors (Donors 1, 2, and 3) during decomposition. Decomposition was assessed as ADD on the *x*-axis and samples were also classified to an overall decomposition stage for each donor at each sampling event (^*^f, fresh; *e*, early; *a*, advanced; and *s*, skeletonized). Minor contributors include data that accounted for <1% of the total diversity within a group.

**Figure 11:**
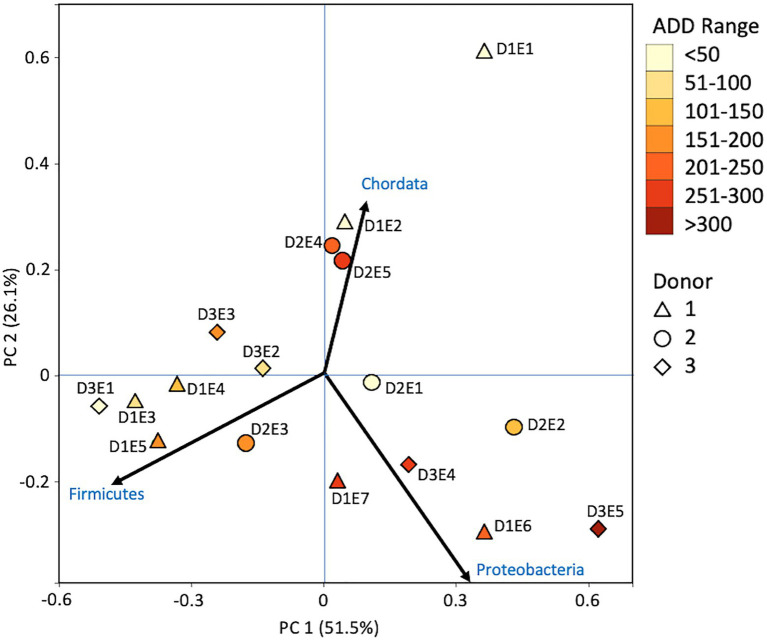
Principal components analysis plot for the proportional data of bacterial and eukaryotic phyla observed from shotgun MetaT sequencing from oral samples of three human donors (Donors 1, 2, and 3) during decomposition. Shading is added to the symbols [triangles for Donor 1 (D1), circles for Donor 2 (D2), and diamonds for Donor 3 (D3)] to show earlier (lighter shading) to later (darker shading) ADD and also times sampled (from E1 to E7 for D1 and E1 to E5 for D2 and D3, indicating first sample to last sample taken).

**Figure 12:**
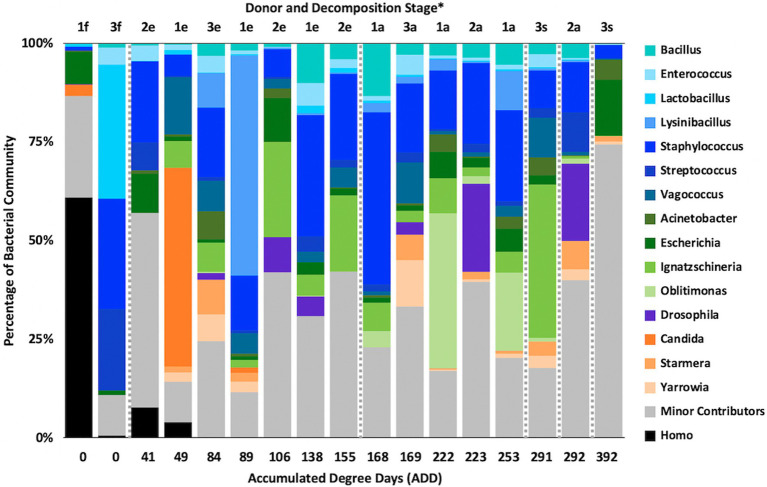
Proportions of bacterial and eukaryotic genera determined from shotgun MetaT sequencing from oral samples of three human donors (Donors 1, 2, and 3) during decomposition. Decomposition was assessed as ADD on the *x*-axis and samples were also classified to an overall decomposition stage for each donor at each sampling event (^*^f, fresh; *e*, early; *a*, advanced; and *s*, skeletonized). Minor contributors include data that accounted for <3% of the total diversity within a group.

**Figure 13:**
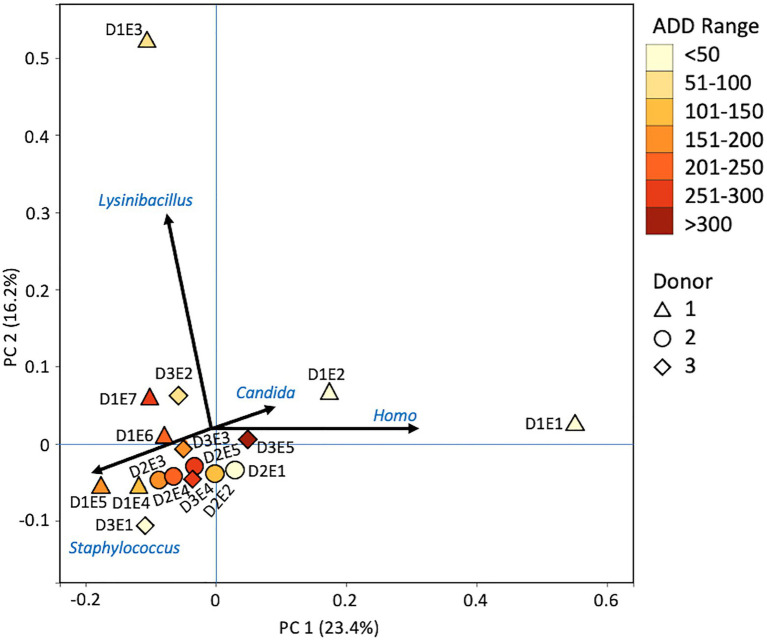
Principal components analysis plot for the proportional data of bacterial and eukaryotic genera observed from shotgun MetaT sequencing from oral samples of three human donors (Donors 1, 2, and 3) during decomposition. Shading is added to the symbols [triangles for Donor 1 (D1), circles for Donor 2 (D2), and diamonds for Donor 3 (D3)] to show earlier (lighter shading) to later (darker shading) ADD and also times sampled (from E1 to E7 for D1 and E1 to E5 for D2 and D3, indicating first sample to last sample taken).

Metatranscriptomic sequencing analyses revealed some eukaryotic RNA throughout the sampling schedule, with Chordata most prevalent early on, Arthropoda increasing in abundance with time (due to sequences related to flies including *Drosophila* and to a lesser extent *Lucilia*, *Rhagoletis*, *Bactrocera*, and *Ceratitis*), Ascomycota fluctuating over time, and Streptophyta being in relatively low abundance most of the time (Figure 10). Bacterial data showed Firmicutes to be common for the early and middle sampling times and Proteobacteria generally being in secondary numbers, except for the last few samples where Proteobacteria generally outnumbered the Firmicutes. Actinobacteria were found in lower numbers in most of the samples, but were once again found primarily in early and late sampling times. The primary drivers of separating the samples using PCA were Chordata, Proteobacteria, and Firmicutes, with the latter two phyla separating samples from the other phyla based on PC1, which accounted for 51.5% of the variation in the dataset and Chordata separating samples along PC2 (Figure 11). The samples did not appear to follow a trend based on donor or ADD. As for the MetaG data, and to a lesser extent the 16S data, minor contributors (accounting for <3% of the data each) were found to be common in total across most of the samples (Figure 12). Human reads were found in the first samples taken for each donor and were more than 50% of the total for the first sample for Donor 1. *Candida* spp. was dominant in one sample (Donor 1, sample 2) while *Drosophila* spp. were observed in about two-thirds of the samples, primarily in the final two samples taken from Donor 2. *Starmera* spp. and *Yarrowia* spp. appeared in the same 10 samples, usually in abundances of <10% of the total. *Staphylococcus* spp. was common throughout the samples while other Firmicutes genera were in lower numbers much of the time. Proteobacteria such as *Escherichia* spp., *Ignatzschineria* spp., and *Oblitimonas* spp. became common and often dominant in the middle to later samples. Human reads and *Candida* spp. (positive relationships for PC1) and *Lysinibacillus* spp. (positive relationship for PC2) appeared to skew three samples in PCA analysis, with *Staphylococcus* spp. showing some influence along the negative axes for the principal components (Figure 13). Most samples were clustered quite close together in the negative quadrant of the plot. As in the 16S and MetaG data, *Lysinibacillus* spp. was found in notably high abundances for MetaT in the third sample for Donor 1.

### Bacterial Isolates

Cultures recovered from the donors spanned the range of ADD and included 46 unique species of bacteria from a total of 69 isolates (Table 2). Species from the Proteobacteria accounted for the largest proportion of the isolates, with 43.5% of the cultures belonging to this phylum. Firmicutes (32.6%), Actinobacteria (19.6%), and Bacteroidetes (4.3%) accounted for the rest of the cultures. Four isolates were recovered from the same donor from multiple sampling times, including *Bacillus cereus*, *Paenarthrobacter nitroguajacolicus*, *Proteus vulgaris*, and *Pseudomonas koreensis*. Eight other cultures were obtained from more than one donor including representatives from *Dermacoccus*, *Morganella*, *Proteus*, *Providencia*, and *Staphylococcus*. Fifteen of the isolates appeared only from early samples (ADD < 100) while another 10 only appeared in late samples (ADD > 250) and 11 of the other 21 appeared in multiple ADD sampling points. Notably, cultures from *Corynebacterium* were obtained from times in which this genus was prevalent in the 16S data from the same samples (Figure 4), while *Pseudomonas* isolates were also recovered from corresponding sampling times that had high levels of *Pseudomonas* sequences present in the community profiles obtained from the sequencing approaches involving 16S (Figure 4) and MetaG (Figure 8). *Acinetobacter* spp. were cultured from samples that showed this genus to be common in the 16S and MetaT sequencing (Figures 4, 12), *Staphylococcus* spp. were common in MetaG and MetaT sampling (Figures 8, 12), and *Bacillus* spp. were common in MetaT sequences (Figure 12). Only five species, *Curtobacterium citreum*, *Dermacoccus nishinomiyaensis*, *Microbacterium saccharophilum*, and *Micrococcus aloeverae*, were not detected in the sequence data. In all, of the 46 isolates, 41 could be matched to species-level reads from at least one of the three sequencing methods, including 11 species that were seen in all three datasets (Table 2). These included *Acinetobacter gerneri*, *B. cereus*, *Comamonas terrigena*, *Kurthia zopfii*, *Lactobacillus paracasei*, *Morganella morganii*, *P. vulgaris*, *P. koreensis*, *Pseudomonas moraviensis*, *Raoutella terrigena*, and *Stenotrophomonas maltophilia*.

**Table 2:** Species of bacteria recovered from three human donors undergoing decomposition at the FOREST facility in Cullowhee, NC including a name for each based on NCBI taxonomy resulting from BLAST analyses, the donor(s) and sampling event(s) that yielded each species, asnd the accumulated degree day(s; ADD) at the time(s) of sampling.

Best NCBI match	Donor	Sampling event(s)	ADD	ADD range	16S rRNA ASVs	MetaG reads	MetaT reads
*Acinetobacter albensis*	1	7	253	251–300	3:6^*^	2:3^*^	--
*Acinetobacter gerneri*	3	4	291	251–300	1:3^*^	2:6^*^	1:2^*^
*Bacillus cereus*	1	2, 4, 6, 7	49–253	0–300	2:2^*^	3:17^*^	3:14^*^
*Bacillus mycoides*	2	4	223	201–250	2:2	3:11^*^	1:1
*Bacillus paranthracis*	2	5	292	251–300	--	3:10	3:11
*Bacillus simplex*	2	5	292	251–300	--	3:10	--
*Comamonas terrigena*	3	5	392	301–400	1:3^*^	2:5^*^	1:2^*^
*Corynebacterium striatum*	3	1	0	0–50	--	1:4^*^	--
*Corynebacterium* xerosis^1^	3	3	169	151–200	1:3^*^	3:7^*^	1:1
*Curtobacterium citreum*^1^	3	1	0	0–50	--	--	--
*Dermacoccus nishinomiyaensis*	1, 3	1, 5	0.3, 168	0–200	--	--	--
*Enterococcus faecalis*	3	1	0	0–50	--	3:17^*^	3:11^*^
*Flavobacterium saccharophilum*^1^	3	5	392	301–400	--	2:2^*^	--
*Hafnia paralvei*	2	2	106	101–150	--	2:6^*^	1:1
*Janthinobacterium lividum*^1^	3	5	392	301–400	--	1:1^*^	1:1^*^
*Kocuria rhizophila*^1^	3	3	169	151–200	--	1:3^*^	--
*Kurthia zopfii*	3	2	84	51–100	2:3^*^	3:12^*^	2:6^*^
*Lactobacillus paracasei*	3	1	0	0–50	2:3^*^	2:7^*^	2:2^*^
*Lactobacillus plantarum*	3	1	0	0–50	1:2	3:15^*^	3:6^*^
*Lysinibacillus fusiformis*	3	4	291	251–300	2:2	3:10	1:1
*Macrococcus canis*	1	5	168	151–200	--	3:9^*^	1:1
*Massilia putida*	3	1	0	0–50	--	1:1	1:1
*Microbacterium lemovicicum*	1	1	0	0–50	1:1	1:2	--
*Microbacterium saccharophilum*^1^	1	1	0	0–50	--	--	--
*Micrococcus aloeverae*	1	2	49	0–50	--	--	--
*Moraxella osloensis*	3	1	0	0–50	--	3:10	2:2
*Morganella morganii*	1, 3	2, 3, 4	84–138	51–150	2:3^*^	3:15^*^	3:10^*^
*Myroides profundi*	1	6	222	201–250	2:3^*^	3:5^*^	--
*Nocardia coeliaca*	2	1	41	0–50	3:4	--	--
*Paenarthrobacter ilicis*	1	2	49	0–50	1:2^*^	--	--
*Paenarthrobacter nicotinovorans*	1, 2	1	0, 41	0–50	1:2	2:2	--
*Paenarthrobacter nitroguajacolicus*^1^^,^^2^	1	2, 3	49, 89	0–100	1:2^*^	--	--
*Proteus terrae*^1^^,^^2^	1, 2	3, 3, 6	89–222	51–250	2:3^*^	3:5^*^	--
*Proteus vulgaris*	1	3, 4	89, 138	51–150	3:6^*^	3:14^*^	2:3^*^
*Providencia alcalifaciens*^1^^,^^2^	2, 3	3	155, 169	151–200	1:1	3:12^*^	3:3
*Providencia vermicola*^1^^,^^2^	2, 3	3	155, 169	151–200	3:6^*^	--	--
*Pseudomonas koreensis*^1^^,^^2^	2	1, 2	41, 106	0–150	1:1^*^	2:2^*^	2:2^*^
*Pseudomonas moraviensis*	2	1	41	0–50	1:1^*^	2:2^*^	1:1^*^
*Pseudomonas weihenstephanensis*^1^	1	6	222	201–250	1:1	1:1	--
*Raoultella terrigena*	3	5	392	301–400	1:2^*^	2:6^*^	1:2^*^
*Serratia liquefaciens*	2	2	106	101–150	--	2:6^*^	--
*Sphingomonas xinjiangensis*^1^	3	3	169	151–200	--	--	--
*Staphylococcus saprophyticus*	1, 2	1, 5	0, 292	0–300	2:4^*^	3:11^*^	--
*Staphylococcus sciuri*	2, 3	2, 3	85, 155	51–200	1:1	3:12^*^	1:1
*Staphylococcus xylosus*	2	4	223	201–250	2:2^*^	3:12^*^	--
*Stenotrophomonas maltophilia*^1^	3	5	392	301–400	1:2^*^	3:11^*^	2:2^*^

The last three columns reflect data for which similarly named taxonomic designations could be found in 16S rRNA gene sequencing, shotgun MetaG sequencing, and shotgun MetaT sequencing. The first value in each of the pairs of numbers separated by a colon in the last three columns includes how many of the three donors yielded a sequence matching the culture shown in the first column while the second number includes how many of the individual samples had a sequence match to that culture.

* indicates that the culture was obtained from a sample taken from the same donor at the same sampling time as one or more of the culture-independent sequencing approaches. (For example, for *Acinetobacter albensis* in the first row, the 16S rRNA ASVs reports 3:6* which means that all three donors had a match to this species from this method and that six of the 17 samples recovered did. The asterisk indicates that the *A. albensis* culture recovered was found on the same day as one of the 16S rRNA ASVs. Where “--” is displayed indicates that a species found in culture was not detected from one of the sequencing methods).

1 culture grown on low nutrient media only.

2 culture grown on low nutrient media and normal strength R2A.

Nearly half of the isolates (*n* = 22) were assessed to match sequences that were recovered from all three donors using at least one of the sequencing techniques (Table 2). For example, putative sequences from *B. cereus*, *C. terrigena*, *K. zopfii*, *L. paracasei*, *M. morganii*, *P. vulgaris*, *P. koreensis*, *P. moraviensis*, *R. terrigena*, and *S. maltophilia* were recovered using 16S, MetaG, and MetaT sequencing. MetaG sequencing was most sensitive in detecting species in multiple sampling events, finding 10 or more records of the same species in the 17 samples and matching 37 of the 46 cultures obtained. MetaT sequencing found >10 matches for the same species in four cases and matched to 24 total cultures while the 16S sequencing matched 29 cultures, but only saw a maximum of 6 of the 17 sampling times reflected in the cultured species, thus suggesting lower sensitivity for detecting cultured bacteria using this method. These numbers are likely reflective of the total sequences generated by each method, with thousands (16S), millions (MetaG), and hundreds of thousands (MetaT) of sequences generated in this study (Supplementary Table 1). Lastly, two species, *B. cereus* and *E. faecalis*, were observed in all 17 of the samples using the MetaG method. These species were also observed in high numbers in the MetaT reads and *B. cereus* was independently cultured at least four times. A 16S ASV was related to *E. faecalis* at the 96.75% homology level (based on 400 bp), so is not reported in Table 2 as a match.

## Discussion

### Overview

To our knowledge, this is the first study to combine 16S sequencing with MetaG and MetaT in exploring the taxonomic diversity in human donors decomposing over time. Many studies have employed next generation sequencing of 16S amplicons from deceased human donors, e.g., Adserias-Garriga et al. (2017) from oral samples; Damann et al. (2015) from bone samples; Hyde et al. (2013) from the mouth and rectum before and after the bloat stage and GI tract after bloat; Hyde et al. (2015) from skin, mouth, and fecal material; Javan et al. (2016) from internal organs, blood, and the buccal cavity; Javan et al. (2017) from the liver and spleen; and Johnson et al. (2016) from the nose and ear cavities. Other microbiome studies based on 16S have included living humans (Costello et al., 2009; Huttenhower et al., 2012), which included samples from the mouth. Several investigations have shown the mouth to contain unique assemblages of bacteria as compared to other locations in the body in both living (Costello et al., 2009; Huttenhower et al., 2012) and deceased humans (Hyde et al., 2013; Javan et al., 2016). Few studies have recently examined microbial cultures from decomposing human remains (Vass, 2001; Janaway et al., 2009) and none appear to have sampled the hard palate as in this study.

### Climate and Other Factors Affecting Decomposition

The donors sampled in this study experienced significantly different temperature regimes during decomposition, which greatly influenced the calculated ADDs (Figure 1) and decomposition state (Table 1). While the seasonal effect as spring progressed may have had an influence on the microbial communities, there were also other compounding factors such as the presence of dentures in Donor 1, which were removed on the first day of sampling, and scavenger activity. When removing these upper dentures, a thick film of unknown origin was found along the hard palate. As for scavenger activity, vultures were spotted during sampling and their activity, which has been corroborated with higher decomposition rates, was observed by video footage, especially for Donor 3 (Bailey, 2020). Vertebrate and arthropod scavengers could have influenced the sequences we detected as they are carriers of microorganisms themselves and through their action can open up deeper tissues for microbial colonization. Due to the lack of barriers, other scavengers were likely active as well. MetaG and MetaT sequencing analyses yielded data linked to avian, rodent, carnivore, insect, and worm taxa (data not shown). Carter et al. (2015) noted faster decomposition rates in pig carcasses in summer vs. winter and also observed reduced fly activity during rainy conditions. Fewer flies and maggots were noted for Donors 1 and 3, for which it was raining at every sampling event. In all three donors, however, fly-associated bacterial taxa including *Ignatzschineria* and *Wohlfahrtiimonas* were present after the first sampling event. In addition to these variables, the time from death to placement in the decomposition facility was 6–8 days and it is unknown what changes to the microbial communities may have taken place during cold storage of the bodies (Table 1). For Donor 2, decomposition would have begun before storage as this individual was dead for 2 days before being discovered. In addition, this donor was obese, a factor that can aid in the acceleration of decomposition (Byard and Tsokos, 2013). Furthermore, the cause of death of the donors was different for each person and originated in organs (heart, stomach, and lungs) distant to the hard palate area that was sampled. Kaszubinski et al. (2020) demonstrated that the manner and cause of death can be linked to the beta-dispersion found in postmortem microbial communities, including those of the mouth. This may be particularly true in cases of heart disease where dysbiosis of the oral cavity has been previously linked to heart conditions (Seymour et al., 2007).

### 16S Phylum Patterns

Based on 16S sequencing, the phyla Firmicutes and Proteobacteria are most often sampled in the human mouth before death, in recently deceased humans, and early in decomposition, while Actinobacteria and Bacteroidetes are often found in appreciable numbers (Huttenhower et al., 2012; Hyde et al., 2013, 2015; Adserias-Garriga et al., 2017). Other locations in the body show similar trends in which these four phyla are dominant in numbers in the early PMI such as for skin (Hyde et al., 2015), liver and spleen (Javan et al., 2017), and ribs (Damann et al., 2015). Our data show high numbers for Firmicutes including in the ADD values up to 50 (fresh stage of decomposition), with proportions from ~25–70% of the total; however, the first sample for Donor 1 consisted of ~75% Actinobacteria (Figure 2), all accounted for by the genus *Rothia* (Figure 4). *Rothia* has been shown to be skewed toward males (Javan et al., 2016) and especially common in early samples during decomposition (Javan et al., 2016; Adserias-Garriga et al., 2017). Donor 1 was the only male in this study and the same *Rothia* ASV found in Donor 1 was also found in the other two donors, with Donor 2 showing a second ASV for this genus. The first sample for Donor 1 showed low diversity (Supplementary Table 1) and otherwise only contained six distinct ASVs, all related to *Lactobacillus* spp.

Middle to later stages of decomposition have shown Firmicutes generally as the most abundant phylum with occasional dominance from Proteobacteria (Hyde et al., 2013, 2015; Adserias-Garriga et al., 2017) and, even more infrequently, Actinobacteria (Hyde et al., 2015). Our data are consistent with these findings for ADD values starting at 84 (early stage of decomposition), in which nine of 13 samples were dominated (>75% of the sequences) or co-dominated (~50% of the sequences) by Firmicutes and five of the 13 samples were dominated or co-dominated by Proteobacteria. In one sample, the second to the last for Donor 3 (291 ADD), Actinobacteria accounted for >50% of the ASVs (Figure 2).

The only other phyla observed for the 16S sequences included Epsilonbacteraeota (Figure 2), which was represented by two ASVs, one found in the first sample for both Donors 2 and 3 and was related to an uncultured *Campylobacter*, and the other found in the last sample for Donor 3 which was related to *Arcobacter*. Both of these genera contain human-associated bacteria, including well known pathogens in *Campylobacter* and bacteria from the oral and other human environments in the case of *Arcobacter* (On et al., 2017). Five unique ASVs from the phylum Fusobacteria were found, all in the first sample for Donor 2. These were related to the genera *Fusobacterium* and *Leptotrichia*, which include anaerobic fermenters and are common in oral and other locations in the body (Gupta and Sethi, 2014). Lastly, one ASV related to an uncultured species of Verrucomicrobia was found in the second sample for Donor 1. This sequence is most closely related to the genus *Akkermansia*, which includes species, found in the human gut that catabolizes mucin (Derrien et al., 2004). Some of these taxa are normally found in the GI tract, but it is not surprising to find them in the oral cavity during decomposition, especially after the bloat stage when gut contents and microbes may purge from these lower sites (Hyde et al., 2013; Adserias-Garriga et al., 2017). In our study, bloat was not observed for any of the donors due to the sampling schedule and limited access to the FOREST facility during our study.

### 16S Genus Patterns

Common genera observed in 16S sequenced from the mouths of human donors sampled during early stages of decomposition include *Streptococcus* (Hyde et al., 2013; Javan et al., 2016; Adserias-Garriga et al., 2017); *Prevotella* and *Veillonella* (Hyde et al., 2013; Javan et al., 2016); *Lactobacillus*, *Bacteroides*, and *Clostridium* (Hyde et al., 2013); *Corynebacterium*, *Escherichia*, and *Actinomyces* (Adserias-Garriga et al., 2017); *Rothia* (Javan et al., 2016; Adserias-Garriga et al., 2017); and *Staphylococcus* and *Haemophilus* (Javan et al., 2016). Our data include ASVs related to all of these genera with *Actinomyces*, *Rothia*, *Clostridium*, *Lactobacillus*, and *Streptococcus* showing notable numbers (~15–45% of the ASVs from at least one sample; Figure 4). Other genera appearing prominently in early samples in our data include *Lysinibacillus*, *Vagococcus*, *Pseudomonas*, and *Ignatzschineria*. These genera are often observed in middle to late decomposition along with *Acinetobacter*, *Enterococcus*, *Erysipelothrix*, and unresolved taxa from the families Bacillaceae and Planococcaceae (Adserias-Garriga et al., 2017). Our data showed *Enterococcus*, *Ignatzschineria*, unknown Bacillales, and unknown Planococcaceae to be common in ADD values beginning at 138 and through 292, suggesting their abundance and perhaps importance in decomposition. Other work has demonstrated that many of the above genera are abundant in the mouths of living humans, including samples specifically taken from the hard palate (Huttenhower et al., 2012), and are also generally found in decomposition of humans (Hyde et al., 2015). Johnson et al. (2016) determined that *Staphylococcus* and *Vagococcus* were good candidates for estimating PMI using a machine learning approach.

As shown above, the ASVs from our 16S sequencing yielded similar genera to those found in the mouths of living people, including some potentially associated with oral diseases (Huttenhower et al., 2012). However, at the level of species, only one of our ASVs was able to be matched to one of the sequences reported in Huttenhower et al. (2012). This was an ASV for *Lactobacillus crispatus*, which may be found in healthy individuals, and was observed to be approximately a quarter of all ASVs identified in the first sample for Donor 1 and for Donor 3. It was not observed in Donor 2 and otherwise only in the second and third samples for Donor 1 as 1–2% of the total sequence records. In all other species shown to exist in the human oral cavity, our records were not able to be directly matched, largely because our output from QIIME2 produced records for uncultured bacteria or taxa with ambiguities. However, in analyzing the sequences for our ASVs against the Expanded Human Oral Microbiome Database (eHOMD; Escapa et al., 2018), another species was able to be matched to one found in the study done by Huttenhower et al. (2012) and was identified as *Rothia mucilaginosa* (100% match), which was assessed to be an opportunistic pathogen. It was only found in the three initial samples taken from all of our donors and in the second sample from Donor 1. For Donor 1, this species accounted for ~70 and ~13% of ASVs sequenced from the first and second sample, respectively. The *R. mucilaginosa* ASV comprised <5% of the total sequences for the initial samples of the other donors, lending some support to the findings of Javan et al. (2016). Commonly occurring genera in our study and the human microbiome (Huttenhower et al., 2012) were also found in the oral cavities of decomposing humans as shown in Adserias-Garriga et al. (2017) and include *Streptococcus*, *Staphylococcus*, *Rothia* (including *R. mucilaginosa*), and *Corynebacterium*. *Streptococcus parasanguinis* was a species identified in this latter study and was also determined to be among our ASVs (at a 100% match to a sequence in eHOMD), being found in a single sample, the first taken from Donor 3, which accounted for ~10% of all ASVs in the sample.

Interestingly, Adserias-Garriga et al. (2017) showed a drop in oral-associated species from as high as ~95% of all species in a donor in early decomposition to <5% of species by the end of decomposition. We examined our ASVs in a similar way and selected 51 sequences to analyze further based on their prevalence in the dataset (defined as accounting for >5% of ASVs collectively across all samples). Of these, 23 ASVs showed >97% sequence identity to eHOMD records and the others fell between 84 and 96.8% similar to 16S sequences in that database (Escapa et al., 2018). For the highly matched sequences, there were 12 species that only appeared early in decomposition from our samples, originating in ADD scores <50. These were *Schaalia* sp. HMT 180, *R. mucilaginosa*, *Prevotella* sp. HMT 313, *Granulicatella elegans*, *L. crispatus*, *L. fermentum*, *L. gasseri*, *L. paracasei*, *Streptococcus parasanguinis*, *Veillonella atypica*, *Neisseria perflava*, and *Haemophilus parainfluenzae*. One poorly matched ASV to the eHOMD records was a *Vagococcus* sequence that accounted for over 38% of the sequences for the second sample for Donor 1 (ADD 49). High sequence identity matches for ASVs from late samples (>ADD 168) to oral taxa were found for *Pseudomonas stutzeri*, *Kluyvera ascorbata*, and *Corynebacterium urealyticum*. Lastly, poorer matches (all <94% sequence identity) to five ASVs were all found in the final sample from Donor 3 (392 ADD) and comprised from 6 to 13% each of the ASVs in that sample. These sequences included an unresolved taxonomic group in the Proteobacteria as well as two genera from this phylum, *Oligoflexus* and *Undibacterium*, as well as two ASVs aligned to the genus *Flavobacterium*. These results show that as decomposition progresses, fewer oral-associated bacteria are detected and an increase in taxa with low matches to the eHOMD database increased, suggesting that these taxa may come from other environments (Adserias-Garriga et al., 2017). Of the late appearing ASVs for Donor 3 with low matches to eHOMD from above, all were found to be most closely related to bacteria isolated from soil, and in one case, from a waterfall.

### MetaG and MetaT Phylum Patterns

The number of numerically abundant phyla that were detected based on sequencing method was similar (7–8 phyla; Figures 2, 6, 10), despite the greater sequencing power of the MetaG and MetaT methods (Supplementary Table 1), which also picked up eukaryotic taxa. However, a total of only seven phyla were sequenced in the 16S work as compared to 66 phyla in the MetaG sequencing and 51 for MetaT. More than half of the phyla for these latter two methods were from bacteria (data not shown). For the bacteria, Actinobacteria, Firmicutes, and Proteobacteria were the most abundant phyla across the three methods, with Actinobacteria showing higher values in the 16S sequences. Unsurprisingly, these three phyla also drove the relationships seen in PCA plots, along with Chordata for the MetaG and MetaT sequence data (Figures 3, 7, 11). These three bacterial phyla accounted for almost all of the cultures as well (Table 2). Based on these results, it would appear these phyla were most numerous and important in the decomposition processes in the FOREST for the donors sampled. As shown previously, this is consistent with other studies using culture or sequencing methods when assessing bacterial diversity *via* 16S in living and decomposing humans (Vass, 2001; Janaway et al., 2009; Huttenhower et al., 2012; Hyde et al., 2013, 2015; Damann et al., 2015; Adserias-Garriga et al., 2017; Javan et al., 2017). What is not apparent, in Figures 3, 7, 11 for the phyla in this study, is any clear trend in the plots based on decomposition time or donor. In Figures 2, 6, 10, one can see temporary increases or “blooms” of some taxa which help to show individual donor differences for one or two sampling events that are reflected in the PCA plots and appear to be outliers in those graphs. These include phyla in the first sample for Donor 1 and the fourth sample for Donor 3 (Actinobacteria), the second and last sample for Donor 3 and the second sample for Donor 2 (Proteobacteria; Figures 2, 3); the first sample for Donor 3 and the third sample for Donor 1 (Firmicutes; Figures 6, 7); and the first sample for Donor 1 (Figures 10, 11; Chordata). These and other individual sample differences may have biological meaning, even if they are not something we can clearly define, based on lack of further data. Below we address some differences seen for the genera sequenced in this study and how some clearer patterns were observed in PCA, especially for early samples, and how some key taxa involved in the decomposition of the donors in this study may have been identified.

### MetaG and MetaT Eukaryotic Genus Patterns

The MetaG and MetaT sequences included data for taxa that would be unexpected in the microflora of human mouths undergoing decomposition, including some members of the phyla Arthropoda, Chordata (non-human DNA and RNA), and Streptophyta (Figures 6, 10). While some of these phyla might be represented in the diet of living humans, it is unclear in many cases why they would increase in abundance through decomposition. For example, the genus *Penaeus* (all matched to *P. vannamei*, the Pacific White Shrimp) was found in all 17 of the MetaG samples and six of the MetaT samples (for Donors 1 and 2 only) and *Olea* (all sequences matched to *O. europaea*, the Common Olive tree) was found in 14 of the MetaG samples and five of the MetaT samples (Donors 1 and 2 only). This suggests a few possibilities, including that all of the donors had consumed foodstuffs with these organisms in them and that DNA or RNA from the food remained in the oral cavities and/or was released from the digestive tract into the mouth during bloat and purge. This is less easy to believe from the MetaT data as it would suggest RNA was present in inactive cellular materials. It could also mean that scavenging organisms carried plant and animal components to the mouths of the donors. It cannot be ruled out that there are as yet unsequenced nucleic acids from many understudied taxa in the sequence databases. Therefore, matches we see for our MetaG and MetaT data may be insufficient to resolve lower ranked taxa. In examining many of the minor contributors to the MetaG and MetaT sequence reads, organisms from other continents that would not be in or near the FOREST site were encountered (data not shown) and it is more plausible to presume we have detected nucleic acids from related taxa as the Kraken2 taxonomic assignment will have a certain degree of false-positives at different confidence settings.

The presence of arthropods such as flies is not surprising as they are mobile and could be drawn to resources provided *via* decomposition (Goff, 2010). Flies and other arthropods could also serve as vectors for the nucleic acids and microbes detected in this study. Eggs and larva from insects could have also been sampled when swabbing the donors as they were observed, but not identified to any taxonomic level (Table 1). Perhaps surprisingly, 32 species allied to *Drosophila* were found in the MetaG reads, none of which were observed in the first sample taken, while 26 presumptive *Drosophila* species were found in MetaT reads in all but the first three samples taken for Donor 1 and the first sample taken for the other donors. To our knowledge, there is no evidence in the literature that shows fruit flies using animal remains for feeding or laying eggs and *Drosophila* spp. are consistently observed in decomposing vegetal matter (Markow and O’Grady, 2008). One laboratory investigation did report feeding fruit flies with irradiated beef and ham as part of their diet in order to detect effects of food sterilization on the genetics of the consumer (Mittler, 1979); so the use of decomposing animal tissue by *Drosophila* cannot be ruled out. In the MetaT dataset, *Drosophila* was often a major contributor to the sequences (Figure 12), but was always a minor contributor in the MetaG data (Figure 8). Donor 2 showed the most insect activity throughout sampling (Table 1) and had the highest values for *Drosophila* RNA sequences. More work will need to be done, including with other MetaT data yet analyzed from this study, to more precisely determine the role of these insects and whether these reads were simply misidentified or truly belong this genus. Two other insect taxa that have been identified as being important in the decomposition of humans, *Lucilia* spp. and *Phormia regina* Meigen, 1826 (Diptera: Calliphoridae; Anderson, 2000) were observed in our donors. *Lucilia cuprina* Wiedemann, 1830 (Diptera: Calliphoridae) nucleic acids were detected using MetaG and MetaT in all three donors, with the majority of the data coming from Donor 2, for which the only records from *P. regina* were also found.

Yeasts such as *Candida* and *Yarrowia* being found in nucleic acid sequences from our samples makes intuitive sense as they are often sampled from living humans (Huseyin et al., 2017) or they could be brought to decomposing bodies *via* flies or by wind dispersal. *Candida* is a well-studied genus of human-associated yeasts and is implicated in many diseases (Huffnagle and Noverr, 2013). We detected *Candida albicans* and *Candida dublinensis* in MetaG sequences in all donors across decomposition, most prominently in Donor 1 (Figure 8). Donor 1 and the last sample for Donor 2 also showed a match for *C. glabrata*. Only Donor 1 had any records for *Candida* in the MetaT sequences (Figure 12) and all three above species were represented, but only in the first three samples. It seems likely that *Candida* was present largely as a commensal of their hosts and, after death, dropped in numbers as decomposition progressed. *Yarrowia* is a widespread yeast and almost all of our sequences in MetaG and MetaT matched to *Yarrowia lipolytica*, which has been cultured from foodstuffs, soil, hydrocarbons including fuels, wastewater, and human clinical samples (Knutsen et al., 2007). The species epitaph refers to the lipase enzymes produced by this yeast, which allows it to subsist on lipids and other complex hydrocarbons, and might explain its appearance throughout decomposition (Figures 8, 12). Lastly, the genus *Starmera*, seen in our MetaG as a minor contributor and more noticeably in our MetaT data (Figure 12), was represented by *Starmera dryadoides*, a yeast of unknown ecology, but has been associated with fig plants infested with insects (Kurtzman et al., 2011), and may have found their way to the donors through an insect or other vector.

### MetaG and MetaT Bacterial Genus Patterns

At the level of genus, many more taxa were seen in the MetaG and MetaT datasets, but most were considered minor contributors in numbers to the overall microbial communities (Figures 4, 8, 12). *Rothia* was influential in separating some of the early samples based on PCA of the 16S data, where it was only found in in the first sample taken for all donors and in the second sample taken for Donor 1 (Figures 4, 5). This genus was also shown to be highly significant in MetaG data where it was only found in in the first (fresh) samples taken from each donor (*p* < 0.001). *Lysinibacillus* was important throughout all three sequencing datasets and influenced the PCA plots in all cases based on its dominance in the third sample for Donor 1. However, this genus was also common in all three methods, appearing in 12, 14, and 13 of the samples taken for the 16S, MetaG, and MetaT sequencing, respectively. *Lactobacillus* spp. played a role in separating the early samples as shown in Figures 4, 5 for the 16S sequencing, but this genus was also present in 16 of the 17 samples taken for both the MetaG and MetaT methods, albeit in decreasing numbers past the first few sampling times. *Oblitimonas* was observed to be influential in later samples, including the MetaG data, in which it helped to separate out the last two samples from Donor 1 from the other samples (Figure 9). It was also found in the MetaT data where it too was common in the final Donor 1 samples. This genus was not seen in any of the 16S sequence data, but was found in 12 and 11 of the MetaG and MetaT samples taken, respectively, but never in the first sample taken from a donor. Two fungal taxa, *Yarrowia* from the MetaG data (Figure 9) and *Candida* from the MetaT data (Figure 13), helped discriminate two samples based on their high abundance. *Yarrowia* accounted for 64% of the community sequences and *Candida* for ~50%, respectively (Figures 8, 12). Overall, there is a pattern for the genera from the earliest sample times (first sample for each donor plus the second sample for Donor 1) to cluster in PCA plots for the 16S and MetaG data, with a weaker pattern seen for the MetaT data. A similar trend was seen at the level of phylum in another study examining oral microbiota during human decomposition (Adserias-Garriga et al., 2017). One bacterial genus, *Comamonas*, which was not observed in high abundance in any of our data, was shown to have a highly significant result based on being most abundant in MetaT sequences from samples taken for Donor 3 that had reached skeletonization (*p* < 0.001).

Five genera were found to be in high numbers (at least 15% of the sequences obtained in a given sample) for all three sequencing methods (Figures 4, 8, 12). These included four from the phylum Firmicutes: *Lactobacillus*, *Lysinibacillus*, *Streptococcus*, and *Vagococcus*, and *Ignatzschineria* from Proteobacteria. The number of samples in which *Lactobacillus* appeared was 5, 16, and 16 for the 16S, MetaG, and MetaT samples, respectively, and these were most numerous in early ADD values, primarily during the fresh decomposition stage. *Streptococcus* was also most prevalent in early decomposition and was found in three of the 16S samples and all of the MetaG and MetaT samples. *Lysinibacillus*, *Vagococcus*, and *Ignatzschineria* were found in 12–16 of the samples from each sequencing method and were most commonly sampled during the early through advanced stages of decomposition. The proteobacterium *Pseudomonas* was common in 16S (12 samples) and MetaG data (16 samples), being present after the fresh stage of decomposition, except in the case of Donor 2, in which decomposition commenced before the body arrived at the FOREST site and *Pseudomonas* was found at the beginning of sampling. This genus was not observed in high numbers for any of the MetaT samples. *Staphylococcus* (Firmicutes) and *Oblitimonas* (Proteobacteria) were common in MetaG (17 and 12 samples, respectively) and MetaT data (17 and 11 samples, respectively), but not in 16S sequences. *Staphylococcus* was most commonly seen in early to advanced stages of decomposition and *Oblitimonas* was observed in advanced stages of decomposition. Cultures were also obtained for species within *Lactobacillus* (two cultures from the fresh stage of decomposition), *Lysinibacillus* (one culture from an early skeleton), *Pseudomonas* (four cultures from fresh to advanced stages of decomposition), and *Staphylococcus* (five cultures from fresh to advanced decomposition; Table 2).

The detection of *Lactobacillus*, *Streptococcus*, and *Staphylococcus* in fresh samples is consistent with these genera being present in the mouths of living people (Huttenhower et al., 2012), the oral environments of decomposing humans (Hyde et al., 2013; Javan et al., 2016; Johnson et al., 2016; Adserias-Garriga et al., 2017), and three *Streptococcus* species have been shown *via* MetaT of the oral microbiome in living humans to be involved in pyruvate fermentation (Jorth et al., 2014). From 10 to 185 distinct taxa at the level of species were found for *Lactobacillus* in the sequences recovered from all three methods in this study, with 16S sequencing giving the lowest number of ASVs (10) as compared to 185 sequence reads for MetaG and 35 for MetaT. Similar patterns existed for species within *Lysinibacillus* (2–37 “species”), *Pseudomonas* (9–495), *Staphylococcus* (8–52), and *Streptococcus* (11–149) with the low end of species-level sequences recovered from 16S ASVs and the high end from MetaG data. *Oblitimonas* was represented by only one sequence type, related most closely to *Oblitimonas alkaliphila*, while *Vagococcus* records were similar in returning 13–15 unique sequences each and *Ignatzschineria* ranged from 5 to 7 sequence types each. Little to no work has been done to tie specific microorganisms to metabolic functioning *in situ* during decomposition. However, all of the above genera and many species in each have been recovered in cultures (Vass, 2001; Janaway et al., 2009) from various sites on the human body during decomposition. No studies have used cultures from the hard palate to describe microbial diversity during decomposition, although many studies have examined the oral microbiota *via* 16S sequencing as referenced above.

### Bacterial Isolates: Insights From Sequencing

The inability to simultaneously determine “who is there” from an environmental sample (e.g., as determined *via* culturing and/or sequencing) and “what are they doing” (e.g., putrefaction involving turnover of organic substrates or nitrogen cycling), often leaves researchers to infer how microorganisms grown in the lab may participate in their native ecosystem. MetaT could provide a vehicle to overcome this so-called “Heisenberg Uncertainty Principle” as it relates to microbial ecology (Madsen, 1998). The inclusion of cultures in this study attempted to directly link living bacteria to MetaT data from functional genes. This work continues and is beyond the scope of this paper; however, to date we have established that many of the cultures we obtained are also represented in 16S sequences, MetaG, and/or MetaT data (Table 2). This suggests that these cultures will be of interest in future work as we further analyze the MetaG and MetaT data for functional genes involved in specific metabolic processes.

At the present time, we can conjecture that the species observed in culture and those most commonly found across the three sequencing methods are likely active in heterotrophic pathways associated with the rich substrates provided by the tissue of the donors. Further metabolic materials come from the microbes themselves as they die and are replaced by others and *via* vectors such as flies and other arthropods that carry materials to the donors, deposit wastes, and leave young to develop in place. Species we found in culture and in MetaT sequencing include *Enterococcus faecalis*, *L. paracasei* and *L. plantarum*, *Staphylococcus sciuri*, and *Lysinibacillus fusiformis*. These species come from well-characterized and metabolically versatile genera, suggesting they probably play an active role in the postmortem microbial community (Trujillo et al., 2015). While the depth of diversity by a few dozen cultures is not as robust as the sequencing methods, these organisms can give us clues as to the metabolic and biochemical processes that happen during decomposition. Comparing their phenotypic traits with MetaT gene transcripts could indicate functions performed by these species during decomposition. Because these species were also generally encountered across sequencing methods, MetaG data might link genome information to 16S sequences, allowing us to find other genes of importance for some of these species.

In general, *Pseudomonas* spp., *Enterococcus* spp., *Staphyloccocus* spp., and *Lactobacillus* spp., e.g., have been extensively studied and are commonly associated with humans (Trujillo et al., 2015). Other species in this study may be less well-known, but have been shown to tolerate wide pH and temperature ranges and can utilize multiple forms of respiration and/or fermentation for metabolism. Some key taxa found across the methodologies in this study are heterotrophs most commonly defined by aerobic metabolic lifestyles. These include *Vagococcus* spp. (Ge et al., 2020), *Lysinibacillus* spp. (Jung et al., 2012), *Oblitimonas alkaphila* (Drobish et al., 2016), and *Ignatzschineria* spp. (Tóth et al., 2007). All four have been shown to grow at a minimum of 10°C and from slightly acidic to alkaline pH and have been recovered from a variety of sample types, including clinical specimens. Their ability to inhabit the seasonally cooler temperatures encountered by our donors concomitant with the higher pH values that accompany decomposition following putrefaction (Vass, 2001) lends credence to their presence in our study. Furthermore, *Ignatzschineria* spp. has been tied closely to human decomposition (Hyde et al., 2015; Adserias-Garriga et al., 2017). We detected 5–7 *Ignatzschineria* species in each sequencing technique, but did not recover any in pure culture.

A common estimate of how many species can be cultured from a given environment is often <1% (Amann et al., 1995) due to a variety of factors including growth media lacking required nutrients (e.g., specific organics, metals, and growth factors), separation of species from commensal or synergistic organisms, and any activation factors for rousing dormant cells or spores. We used low concentrations of organics in the media to discourage some fast-growing bacteria. We also incubated samples aerobically at room temperature. Many bacteria and yeasts that were abundant in the samples based on sequencing methods were not obtained in culture. In some cases, this was likely because the organisms were anaerobes (e.g., *Clostridium* spp.), which have been shown to be prevalent in other work (Hyde et al., 2013, 2015; Javan et al., 2017). Perhaps *Clostridium* is not as ecologically important in the mouths of donors exposed to air; indeed, it is not commonly reported in the mouths of living humans (Huttenhower et al., 2012; Escapa et al., 2018). Other taxa that escaped cultivation included species from *Ignatzschineria*, *Oblitimonas*, *Vagococcus*, *Candida*, and *Yarrowia*. Each of these genera should be cultured readily using selective media, perhaps informed by MetaG and MetaT data that will be analyzed in future work. Swabs were preserved for each donor at each sampling time, so this work could be done and would go a long way toward understanding the microbial assemblages associated with decomposition.

## Conclusion

While the overall fluctuations of taxa observed in this study were not unlike what has been observed in similar studies, there was no apparent relationship between the length of the PMI and the bacterial community present at a given time. However, taxa from the normal human oral microbiome before death (e.g., *Lactobacillus*, *Streptococcus*, *Rothia*, and *Candida*) did give way to other genera as time progressed, e.g., *Lysinibacillus*, *Vagococcus*, *Ignatzschineria*, and *Yarrowia*. A distinction could often be made between the community from fresh vs. later sampling times, but the finer resolution required for PMI estimation was not elucidated. There were many factors that may have influenced the data in this study, including temperature, humidity, sample size, scavenger activity, and donor sex, weight, and cause of death. However, because the taxa in this study were similar to those of other studies, it is possible that, if incorporated into a comprehensive dataset, a better predictability between PMI and the thanatomicrobiome could be discerned. The approach of integrating culturing techniques alongside whole MetaG and MetaT sequencing to further examine the bacterial communities in question yielded promising data for discerning the specific roles of each taxa in human decomposition. Notably, most of the cultures were found to match data from at least one sequencing technique while 11 cultures were found to match donor and sample times from all three sequencing techniques. We will continue this work to examine prevalent functional genes from many of these species in the MetaG dataset and attempt to link the species we have cultured to expressed genes from the MetaT data. While it is beyond the scope of this paper to include such data, it offers exciting avenues of study to help the field of the postmortem microbiome to advance.

## Data Availability Statement

All high throughput reads have been assigned accession numbers ERS6384843 and ERS6384845-ERS6384860 in the European Nucleotide Archive. The culture 16S sequences are in GenBank and have been given accession numbers MZ067725-MZ067793.

## Author Contributions

EA and SO’C designed the experiment and oversaw the culturing and characterization of the bacterial isolates generated in this work, and EA, KZ, and SO’C received funding for the study. KZ provided access to the human donors, shared field notes throughout decomposition, and gave expert advice about decomposition processes. EA collected all samples and extracted the nucleic acids used in the study. AC conducted all of the 16S, MetaG, and MetaT sequencing and ran the bioinformatics analyses. AC, EA, and SO’C analyzed the data following bioinformatics analysis and SO’C wrote the majority of the manuscript. All authors contributed to the article and approved the submitted version.

### Conflict of Interest

The authors declare that the research was conducted in the absence of any commercial or financial relationships that could be construed as a potential conflict of interest.

## Footnotes

^1^https://www.ncdc.noaa.gov/

^2^http://www.github.com/biobakery/kneaddata

^3^www.uniprot.org/uniref
