# Supplementary material for: Characterization of Bacterial Community Dynamics of the Human Mouth Throughout Decomposition via Metagenomic, Metatranscriptomic, and Culturing Techniques
Source: Front Microbiol. 2021 Jun 7;12:689493. doi: 10.3389/fmicb.2021.689493 (PMC8215110; doi:10.3389/fmicb.2021.689493)
Supplement: Supplementary file 1 [file Table_1.DOCX]

**Supplementary Table 1.** Descriptive data for three human donors undergoing decomposition at the FOREST facility in Cullowhee, NC including the number of sequences and distinct ASVs generated by 16S rRNA gene sequencing, shotgun metagenomic sequencing (MetaG), and shotgun metatranscriptomic (MetaT) sequencing methods. The Shannon diversity index (H) and Shannon evenness value (E_H_) is reported for each sample. D = Donor and E = sampling event with accumulated degree days (ADD) reported for each donor/sampling event. E_H_ was statistically significant for D1 versus D2 for shotgun metagenomic data and for H for metatranscriptomic data (one-way ANOVA, p = 0.02 for both comparisons; Bonferroni post-hoc test, p < 0.05 for both). The mean number of distinct ASVs for Donor 1 was also found to be significantly lower than those sequenced for Donors 2 and 3, with no difference between these donors (ANOVA p = 0.0079; Bonferroni p < 0.05). No other statistically significant results were found based on donor or ADD for any of the other data.

| **Sample** | **ADD** | **16S rRNAs Sequenced** | **Distinct 16S rRNA ASVs** | **H** | **E_H_** | **MetaG Reads** | **Distinct Groups at Species Level** | **H** | **E_H_** | **MetaT Reads** | **Distinct Groups at Species Level** | **H** | **E_H_** |
| --- | --- | --- | --- | --- | --- | --- | --- | --- | --- | --- | --- | --- | --- |
| D1E1 | 0 | 2981 | 7^a^ | 1.61 | 0.57 | 271614 | 195 | 3.30 | 0.43^a^ | 111090 | 151 | 2.96^a^ | 0.41 |
| D1E2 | 49 | 5266 | 44^a^ | 2.93 | 0.52 | 130553 | 183 | 4.31 | 0.57^a^ | 47134 | 46 | 3.43^a^ | 0.62 |
| D1E3 | 89 | 8594 | 23^a^ | 0.86 | 0.20 | 3950753 | 1676 | 3.44 | 0.32^a^ | 794522 | 253 | 3.37^a^ | 0.42 |
| D1E4 | 138 | 3922 | 36^a^ | 3.36 | 0.65 | 2643698 | 3466 | 6.42 | 0.55^a^ | 183352 | 194 | 4.97^a^ | 0.65 |
| D1E5 | 168 | 6584 | 35^a^ | 2.21 | 0.44 | 2545610 | 3343 | 6.73 | 0.57^a^ | 633192 | 339 | 4.28^a^ | 0.51 |
| D1E6 | 222 | 4261 | 31^a^ | 2.52 | 0.51 | 2991373 | 2237 | 3.48 | 0.31^a^ | 1782678 | 443 | 2.71^a^ | 0.31 |
| D1E7 | 253 | 6138 | 38^a^ | 3.19 | 0.60 | 3885496 | 3283 | 4.85 | 0.42^a^ | 237732 | 179 | 4.49^a^ | 0.60 |
| D2E1 | 41 | 4893 | 61^b^ | 4.72 | 0.79 | 1024669 | 1050 | 6.71 | 0.67^b^ | 84837 | 145 | 4.34^b^ | 0.60 |
| D2E2 | 106 | 5049 | 34^b^ | 2.18 | 0.42 | 2018483 | 912 | 6.27 | 0.64^b^ | 430263 | 265 | 4.77^b^ | 0.59 |
| D2E3 | 155 | 3598 | 74^b^ | 4.75 | 0.77 | 2116593 | 3043 | 7.34 | 0.63^b^ | 404891 | 492 | 5.40^b^ | 0.60 |
| D2E4 | 223 | 4566 | 63^b^ | 3.30 | 0.56 | 3072923 | 1492 | 7.23 | 0.69^b^ | 235997 | 280 | 5.24^b^ | 0.64 |
| D2E5 | 292 | 2759 | 61^b^ | 3.18 | 0.54 | 3022553 | 2027 | 7.49 | 0.67^b^ | 110502 | 142 | 5.06^b^ | 0.71 |
| D3E1 | 0 | 5484 | 28^b^ | 3.69 | 0.77 | 1500361 | 474 | 5.14 | 0.58^a,b^ | 141260 | 57 | 3.54^a,b^ | 0.61 |
| D3E2 | 84 | 8450 | 65^b^ | 3.14 | 0.52 | 3269969 | 2336 | 7.11 | 0.64^a,b^ | 180215 | 198 | 5.03^a,b^ | 0.66 |
| D3E3 | 169 | 4098 | 74^b^ | 4.58 | 0.74 | 3194404 | 3616 | 7.57 | 0.64^a,b^ | 230634 | 220 | 5.08^a,b^ | 0.65 |
| D3E4 | 291 | 4094 | 62^b^ | 2.50 | 0.42 | 1984543 | 1198 | 3.04 | 0.30^a,b^ | 182521 | 154 | 4.52^a,b^ | 0.62 |
| D3E5 | 392 | 1229 | 53^b^ | 4.43 | 0.77 | 2598398 | 6976 | 8.91 | 0.70^a,b^ | 699764 | 746 | 5.61^a,b^ | 0.59 |
